# Supplementary material for: Systematic review of lay consultation in symptoms and illness experiences in informal urban settlements of low-income and middle-income countries
Source: BMJ Open. 2021 Dec 21;11(12):e050766. doi: 10.1136/bmjopen-2021-050766 (PMC8693092; doi:10.1136/bmjopen-2021-050766)
Supplement: Supplementary data [file bmjopen-2021-050766supp001.pdf]

Supplementary A: PRISMA checklist

Section/topic	#	Checklist item	Reported on page #
**TITLE**			
Title	1	Identify the report as a systematic review, meta-analysis, or both.	1
**ABSTRACT**			
Structured summary	2	Provide a structured summary including, as applicable: background; objectives; data sources; study eligibility criteria, participants, and interventions; study appraisal and synthesis methods; results; limitations; conclusions and implications of key findings; systematic review registration number.	2
**INTRODUCTION**			
Rationale	3	Describe the rationale for the review in the context of what is already known.	3-4
Objectives	4	Provide an explicit statement of questions being addressed with reference to participants, interventions, comparisons, outcomes, and study design (PICOS).	4
**METHODS**			
Protocol and registration	5	Indicate if a review protocol exists, if and where it can be accessed (e.g., Web address), and, if available, provide registration information including registration number.	5
Eligibility criteria	6	Specify study characteristics (e.g., PICOS, length of follow-up) and report characteristics (e.g., years considered, language, publication status) used as criteria for eligibility, giving rationale.	5
Information sources	7	Describe all information sources (e.g., databases with dates of coverage, contact with study authors to identify additional studies) in the search and date last	5

		searched.	
Search	8	Present full electronic search strategy for at least one database, including any limits used, such that it could be repeated.	5
Study selection	9	State the process for selecting studies (i.e., screening, eligibility, included in systematic review, and, if applicable, included in the meta-analysis).	5-6
Data collection process	10	Describe method of data extraction from reports (e.g., piloted forms, independently, in duplicate) and any processes for obtaining and confirming data from investigators.	6
Data items	11	List and define all variables for which data were sought (e.g., PICOS, funding sources) and any assumptions and simplifications made.	N/A
Risk of bias in individual studies	12	Describe methods used for assessing risk of bias of individual studies (including specification of whether this was done at the study or outcome level), and how this information is to be used in any data synthesis.	N/A
Summary measures	13	State the principal summary measures (e.g., risk ratio, difference in means).	N/A
Synthesis of results	14	Describe the methods of handling data and combining results of studies, if done, including measures of consistency (e.g., I^2) for each meta-analysis.	6-7
Risk of bias across studies	15	Specify any assessment of risk of bias that may affect the cumulative evidence (e.g., publication bias, selective reporting within studies).	N/A
Additional analyses	16	Describe methods of additional analyses (e.g., sensitivity or subgroup analyses, meta-regression), if done, indicating which were pre-specified.	N/A
**RESULTS**			
Study selection	17	Give numbers of studies screened, assessed for eligibility, and included in the review, with reasons for exclusions at each stage, ideally with a flow diagram.	7
Study characteristics	18	For each study, present characteristics for which data were extracted (e.g., study size, PICOS, follow-up period) and provide the citations.	8-10

Onuegbu C, *et al. BMJ Open* 2021; 11:e050766. doi: 10.1136/bmjopen-2021-050766

Risk of bias within studies	19	Present data on risk of bias of each study and, if available, any outcome level assessment (see item 12).	N/A
Results of individual studies	20	For all outcomes considered (benefits or harms), present, for each study: (a) simple summary data for each intervention group (b) effect estimates and confidence intervals, ideally with a forest plot.	N/A
Synthesis of results	21	Present results of each meta-analysis done, including confidence intervals and measures of consistency.	13-17
Risk of bias across studies	22	Present results of any assessment of risk of bias across studies (see Item 15).	N/A
Additional analysis	23	Give results of additional analyses, if done (e.g., sensitivity or subgroup analyses, meta-regression [see Item 16]).	N/A
**DISCUSSION**			
Summary of evidence	24	Summarise the main findings including the strength of evidence for each main outcome; consider their relevance to key groups (e.g., healthcare providers, users, and policy makers).	17-19
Limitations	25	Discuss limitations at study and outcome level (e.g., risk of bias), and at review-level (e.g., incomplete retrieval of identified research, reporting bias).	19
Conclusions	26	Provide a general interpretation of the results in the context of other evidence, and implications for future research.	17-19
**FUNDING**			
Funding	27	Describe sources of funding for the systematic review and other support (e.g., supply of data); role of funders for the systematic review.	19

Supplementary B: Search terms

		Search terms: used in Medline, PsychInfo, ASSIA, CINAHL Web of Science and Scopus
#5	Combination of terms	#1 AND #2 AND #3 AND #4
#4	Low- and Middle-Income Countries	(afghanistan OR "burkina faso" OR burundi OR "central african republic" OR chad OR "democratic republic of the Congo" OR "eritea" OR "ethiopia" OR "gambia" OR "guinea" OR "guinea-bissau" OR "haiti" OR "democratic people's republic of korea" OR liberia OR madagascar OR malawi OR nepal OR niger OR rwanda OR "sierra leone" OR somalia OR "south sudan" OR syria OR tajikistan OR tanzania OR togo OR uganda OR yemen OR angola OR bangladesh OR bhutan OR bolivia OR cambodia OR "cabo verde" OR cameroon OR comoros OR congo OR "cote d'Ivoire" OR "cape verde" OR "ivory coast" OR djibouti OR egypt OR "el salvador" OR ghana OR honduras OR india OR indonesia OR kenya OR "Krygyz republic" OR laos OR lesotho OR mauritania OR micronesia OR moldova OR mongolia OR morroco OR myanmar OR ni caragua OR nigeria OR pakistan OR "papua new guinea" OR philippines OR "sao tome and principe" OR senegal OR "solomon islands" OR sudan OR swaziland OR timor-leste OR tunisia OR ukraine OR uzbekistan OR vanuatu OR vietnam OR "west bank or gaza" OR zambia OR zimbabwe OR albania OR algeria OR "american samoa" OR argentina OR armenia OR azerbaijan OR belarus OR belize OR "bosnia and herzegovinia" OR botswana OR brazil OR bulgaria OR china OR colombia OR "costa rica" OR cuba OR dominica OR "dominican republic" OR "equatorial guinea" OR ecuador OR fiji OR gabon OR georgia OR grenada OR guatemala OR guyana OR iran OR iraq OR jamaica OR jordan OR kazakhstan OR kosovo OR lebanon OR libya OR macedonia OR malaysia OR maldives OR mauritius OR "marshall islands" OR mexico OR montenegro OR namibia OR nauru OR paraguay OR peru OR romania OR russia O R "russian federation" OR samoa OR serbia OR "sri lanka" OR "saint vincent and grenadines" OR "south africa" OR "saint lucia" OR suriname OR thailand OR tonga OR turkey OR turkemenistan OR tuvalu OR venezuela OR benin OR kiribati OR mozambique OR mali)
#3	Informal urban settlements	(slum* OR "inner city*" "ghetto*" OR "informal settlement*" OR "shanty town*" OR "poverty area*"))

#2	Illness behaviours	("patient acceptance of health care" OR "health knowledge" OR "health attitude*" OR "health practice*" OR "care seeking" OR "healthcare seeking" OR "critical pathways" OR "pathway to care" OR "patient care planning" OR "advance care planning" OR "lay symptom evaluation" OR "symptom appraisal" OR "help seeking behaviour*" OR "self care" OR health OR "child health" OR "health status" OR "social determinants of health" OR "family health" OR "minority health" OR "health behaviour*" OR "attitude to health" OR "health seeking" OR "help seeking" OR "illness attribution*" OR "treatment seeking" OR "treatment seeking behaviour*" OR "health service utilisation" OR "access to health care" OR "sick role" OR "illness behaviour*" OR "information seeking behaviour*" OR care OR "health services accessibility")
#1	Lay consultation behaviours<br><br>Social networks	("health advice" OR "social support" OR "interpersonal relation*" OR "social network*" OR family OR "social environment" OR "community network*" OR "social capital" OR community OR "residence characteristic*" OR kin OR friend* OR "informal network*" OR "lay consultation*" OR "lay consultation network*" OR "lay referral*" OR "lay referral network*" OR "strong tie*" OR "weak tie*" OR "social determinants of health" OR "network structure" OR "personal network*" OR parent* OR advice-seeking OR advice OR "lay counselling" OR "social interaction*" OR neighbour* OR neighbourhood OR "Birth companion*" OR doulas OR buddies OR "community health agent*" OR "community health advocate*" OR "community health educator*" OR "community health outreach worker*" OR "community health representative*" OR "community health volunteer*" OR "community health worker*" OR "family health worker*" OR "natural helper*" OR "lady health worker*" OR "lay advisor*" OR "lay counsellor*" OR "lay carer*" OR "lay health educator*" “OR lay health advisor*" OR "lay health leader*" OR "lay health promoter*" OR "lay health volunteer*" OR "lay health worker*" OR "lay helper*" OR "lay supporter*" OR "peer counsellor*" OR "intergenerational relation*" OR "social support" OR "lay consultant*")

Supplementary C: Data extraction form

1	Publication details	Allinger & Zamora (2004) Herbal Remedies in a Nicaraguan Barrio. *Journal of Transcultural Nursing*,15 (4), 278-282
	Main study objectives/research question	To examine the use of herbal remedies in treating common illnesses.
	Study area/ Country of study	- The sample for this study of herbal medicine came from the population of the barrio, where 1,674 people live in 270 households. There was an average of 6.2 persons per household living in dwellings of approximately 15 by 15 feet.<br>- Nicaragua
	Study design	-Interview using a seven-item open-ended interview schedule was developed by the first author to assess the prevalence of herbal remedy use.
	Study population and sample recruitment	- The focus of the first part of the interview was on who had been ill in the household in the last 3 months, their symptoms, and their treatment. Probes were used to ask if any herbal remedies were used. In the second part of the interview, the participants were asked about the remedies they used to prevent illness and their sources of information about the herbal remedies.<br>- In the field, random sampling by placing the block numbers in a hat and drawing numbers was used to select 8 of the 14 blocks within the barrio. Subsequently, four households in each block were selected using a systematic random sample of every fifth household. Interviews were conducted with an adult in each household if he or she gave informed consent. The final sample included 27 respondents. There were 25 women and 2 men in the study.
	Data collection tools, process and analysis	-The design of the study was triangulated with both quantitative and qualitative aspects. Observations during several years of prior ethnographic fieldwork in the barrio revealed that herbal remedies were used for a variety of conditions. Therefore, in the spirit of cultural consensus (Romney, Weller,& Batchelder, 1988), a seven-item open-ended interview schedule was developed by the first author to assess the prevalence of herbal remedy use.<br>-Content analysis was used to develop categories from the responses to each of the questions.The frequencies of categories were examined, and differences in age and education based on use of herbal remedies were calculated. The transcription from the interview with the herbalist was also a data source and was content analyzed with the aid of The Ethnograph (Seidel,1998). One of the limitations of the study was that there was no exclusion of participants based on illness.
	Findings relevant to the review	The sources of suggestions for remedies were grandmother, mother, older people, friends, doctor, and midwife. Almost 30% said that no one had told them about the remedies; they just knew about them, indicating that the use of herbal remedies was embedded in the culture.
2	Publication details	Amuyunzu-Nyamongo & Nyamongo (2006) Health Seeking Behaviour of Mothers of Under-Five-Year-Old Children in the Slum Communities of Nairobi, Kenya *Anthropology & Medicine*, 13:1, 25-40.
	Main study objectives/research question	What actions do mothers take during childhood illnesses and what can be done to strengthen the management of childhood illnesses in the informal settlements?

	Study area/ Country of study	-This paper is based on data collected in four slum communities of Nairobi City, namely, Kawangware, Korogocho, Viwandani and Njiru1 in 2002. The four sites were selected for this study mainly because they formed the sites where the African Population and Health Research Centre (APHRC) had implemented a pilot demographic surveillance system. The four sites have a combined population of about 230,000 people (GOK 2001) of which children under five years old account for 15%, while women aged 15–49 years account for 26%, with minor variations between the slum communities.<br>-Kenya
	Study design	Qualitative design using Indepth interviews
	Study population and sample recruitment	Women whose under-five children had been sick in the previous three months.<br>-In order to get detailed information about child health, we conducted in-depth interviews (IDIs) with 62 mothers from the four study communities (14–17 per site). The women were identified from the pilot demographic surveillance system (DSS) database and the interviewers were given identification information on women whose under-five children had been sick in the previous three months, to collect more details on the conditions and views regarding the causes, the course of action taken and healthcare options used.
	Data collection tools, process and analysis	Eight social science graduates with experience in qualitative research were recruited and trained for data collection. In order to get detailed information about child - health, we conducted in-depth interviews (IDIs) with 62 mothers from the four study communities (14–17 per site). Although we inquired on illness that affected children under five years within the last three months, the mothers reported that the children had, on average, at least two episodes per month. The mothers thus tended to report on current illnesses or those that had taken place within two to three weeks. The study focused on health conditions that cause high morbidity in the area, namely, diarrhoea, pneumonia, cough, tuberculosis and malnutrition. The IDIs lasted on average 40 minutes and were conducted in either Kiswahili or English depending on the language the respondent felt comfortable with. All the interviews were tape-recorded and transcribed.<br>The data have been analysed thematically through reading all the transcripts. Data are provided on what course of action the mothers took when their children were taken ill, length of delay and reasons. Other questions asked included who made the decision to seek care, the frequency of illness among their children and their views regarding the health facilities in their areas
	Findings relevant to the review	Nineteen of the 28 mothers reported making decisions, on four instances the child’s father decided in two both the mother and father decided, the action to be taken, in two neighbours took the decisions due to the urgency of the conditions (one was an acute pneumonia case in the absence of the -, mother), while in one case the child’s grandmother made the decision (the mother of this child was a single mother who lived with her mother). In one situation the child’s mother made the decision assisted by her sisters.<br>- There was reluctance expressed in seeking care in ill-health conditions that were viewed to be chronic. The mother who reported her child suffered from malnutrition said that the problem had lasted three months. She had taken her to local clinics and had gone for prayers where she was, in addition to receiving treatment, advised to give the child porridge. Eventually neighbours told her to take the child to hospital for appropriate care.
3	Publication details	Angeli *et al,(* 2018) Socio-Cultural Sustainability of Private Healthcare Providers in an Indian Slum Setting: A Bottom-of-the-Pyramid Perspective *Sustainability, 10*, 4702

	Main study objectives/research question	Qualitative research was used to gather a deeper understanding of the drivers of choice among BOP patients by employing grounded theory methods to find out BOP patients' main concerns and social processes in making consumption choices.
	Study area/ Country of study	The research is set in the city of Ahmedabad, in the state of Gujarat, India
	Study design	Interviews were conducted to capture the motivations, beliefs, and perceptions of the informants. The two research methods offered complementary perspectives on BOP patients' consumer behavior. Indeed, while quantitative methods revealed the behavior of the consumer group regarding its preferences between private and public hospitals, qualitative methods unveiled the individual consumer's choice decision and motivations
	Study population and sample recruitment	BOP patients in selected slum areas of Ahmedabad. The informants were recruited using two eligibility criteria. Firstly, they had to live in a slum area; secondly, they, their children, or their parents must have visited a private or a public hospital in the last two years.<br>-We used a saturation-driven sampling approach, where we continued recruiting informants until no new information could be obtained from them. This method resulted in a sample of 21 informants, with a sample size in line with prior qualitative research work, particularly with qualitative studies conducted in slum settings. The informants were recruited using two eligibility criteria. Firstly, they had to live in a slum area; secondly, they, their children, or their parents must have visited a private or a public hospital in the last two years. Informants were initially recruited through personal contacts of one of the co-authors. Then, snowball sampling allowed us to recruit additional informants by requesting the earlier informant store commend others who met the eligibility criteria. This approach facilitated access and trust formation while eliminating non-response bias. This technique is deemed suitable to ensure theoretical representativeness of the sample and is argued to be particularly effective in "hard-to-reach" settings.
	Data collection tools, process and analysis	-The interviews aimed to get a deep understanding of the perspective of BOP consumers on private and public hospitals, with specific reference to their decision-making process and the perceived socio-cultural gap with the services offered by the hospitals. The interview protocol was developed in consultation with expert practitioners and academics and was piloted with a purposive sample of slum-dwellers before finalizing it. The final interview guide covered questions about the informants' personal situation, their knowledge and opinion of the hospitals, the transportation used, the hospital treatment received, the payment method, and the main drivers of their choices. The interviews were primarily conducted in Hindi, a language spoken in most of India, with the help of two translators who were not only proficient in both English and Hindi, but also had basic knowledge of Gujarati, a language widely spoken in the state of Gujarat, since not all inhabitants of Ahmedabad speak Hindi fluently. Informants were interviewed in their residential area to ensure the atmosphere was comfortable. A modest gift was given to each participant. An activity and experience log was kept for each day of the interviews. The interviews were recorded, and the English translation provided by the translator was transcribed.<br>-The interviews' transcripts were coded through thematic analysis , which facilitates the tasks of identifying, inferring, and recording patterns and groups of meaning within collected data. The three-step approach suggested by Gioia and colleagues was adopted, which resembles the phases of open and axial coding described earlier by Strauss and Corbin. In the first step,

		transcripts were coded with the aim of being as adherent as possible to the voices and interpretations of the respondents. This process has the advantage of stimulating the researcher's reflective awareness of her/his own biases and assumptions. Through this first process of open coding, 280 segments were coded along 32 first-order codes. In the second step, we took a more knowledgeable stance and started abstracting from the empirical to the theoretical level by identifying similarities across first-order codes. This process of axial coding led us to cluster our first-order codes into nine more general and abstract second-order themes. In the last step, similarities across second-order themes were ascertained, which allowed for subsequent grouping of second-order themes into three abstract aggregate dimensions. Throughout the steps, we engaged in a cycling, iterative process of comparison, aggregation, and re-aggregation of concepts, themes, and higher-order categories. Comparisons with constructs drawn from extant literature helped us identify discrepancies and similarities between our findings and prior research, which we, however, kept at a distance, to maintain a state of "semi-ignorance". Overall, the analytical approach adopted here can be considered as abductive, not entirely informed by prior, preconceived ideas, nor fully guided by empirical findings only.
	Findings relevant to the review	Provider Choice as a Socio-Cultural Process: The choice of healthcare provider appears to be deeply influenced by the patients' socio-cultural circumstances at the moment of illness, by the established trust toward specific providers, and by the opinion and experiences of family members, friends, and community-dwellers at large concerning healthcare services. Among these socio-cultural circumstances, family obligations hold an important role, as patients living in resource-constrained settings often must restrain from seeking healthcare to avoid heavy repercussions of their absence on their families' livelihood, safety, or order (first-order theme "family and work obligations"). As one respondent told us, I am the eldest sister of three sisters—all unmarried. So, I have the responsibilityof the house, and of the two younger sisters as well. If I go for the operation, if something happens to my life,who will take care of my house and my two younger sisters? I'll have some care when my family problems will come down. At that time, I'll get operated. (Jamuna). Trust and familiarity toward the hospital and specific physicians emerge as important factors in guiding the choice of healthcare provider. BOP patients report high levels of loyalty toward doctors and institutions, which are triggered by positive personal experiences, or by recounting of family or community members (first-order theme "familiarity and trust"). Also, respondents report a personal connection with healthcare providers as important to cement the feeling of trust (first-order theme "personal connections"). A respondent notes, So, from childhood days, father and sons, everyone is going to this hospital, and they get cured by the prescribed medicines; now, they have an emotional touch and good experience. Because we are emotional people ... you know. Yeah, so an emotional connection with this hospital. (Abhijit). Familiarity with the doctor and trust are particularly valuable in the examined setting since people living in poor conditions often feel intimidated by doctors and hospitals. The disadvantaged conditions of BOP patients, their low level of education and, hence, their limited understanding of medical science may instill irrational fears (first-order themes "low level of education influencing perception of care" and "extreme poverty"). For example, one respondent recalled running away from a hospital to avoid treatment that seemed suspicious or too experimental. Author: So he's scared about the VS hospital because he fears that they will treat him badly. Translator: Yeah. Not badly, but, ehm, he fears that they want to experiment with his body. Author: Because he is poor? Translator: Yeah obviously because he is poor. (Akshay) It is, therefore, not surprising that healthcare-seeking decisions do not rely only on personal assessments, but also heavily on the opinions and experiences of the community as a whole, in terms of family members, neighborhoods, and kin (second-order theme "provider

		choice arising from shared decision making", combining first-order themes "word-of-mouth", "advice from family members", "mutual, collective, community-level choice", and "family members' support during treatment". The following excerpt is illustrative of this point: So, everyone has said that Dr. Umesh is good, his treatment is good. We knew that he is good, we are familiar with him and he is very nice. A strong advantage is that he straightaway starts the treatment because he is familiar with the local people here. So he doesn't always ask to deposit the advance amount first. Everybody really likes him, and he's a specialist for children. Most of the people who go to him get cured by taking the prescribed medicines. (Rupa) Another important dimension was the doctor's friendliness (first-order theme "friendliness of the doctor"). Several of our respondents noted that private doctors, more often than doctors in public hospitals, tended to show friendliness and familiarity and to establish long-term relationships with the community. I think the Latesia[privatehospital] is good because the doctor doesn't think the patient as a patient, he himself seems like they are my near ones and dear one. The moment I was scared to go for angioplasty because I was in a very bad condition; the doctors said it's curable,think of me as your brother or nas your father. At that time, I thought that he is not my brother my father but he is like a god for me. Because when I was done with angioplasty, I was perfectly alright, and he was the one who encouraged me and gave all the motivation and support that I was looking for at that point of time. So I have great respect for him. (Jamuna) Overall, based on word-of-mouth and familiarity, private providers seem to be strongly preferred by BOP patients—a point we return to in the next section. One of our respondents recalled that, So, they went twice to the government hospital, but their medical problem wasn't cured, so they decided to visit Bhavanbhai [private hospital], because she's very popular, and they don't want to get operated in a government hospital. There, a couple of people died because they believe that most of the people in government hospitals are trainees, they are not the actual doctors. So, they try to keep themselves away; when it comes to the operation, they are scared to get it done at the government hospital. (Preeti).
4	Publication details	Bhandari et al. (2002) Pathways to Infant Mortality in Urban Slums of Delhi, India: Implications for Improving the Quality of Community- and Hospital-based Programmes. *Journal of Health, Population and Nutrition,* 20(2):148-155
	Main study objectives/research question	The study aimed at obtaining insights into the processes underlying infant deaths to help identify preventive interventions which may bring down infant mortality rates further.
	Study area/ Country of study	The study was conducted in two urban slums in Delhi, India, between February 1995 and August 1996
	Study design	Verbal autopsies were conducted at home, using a semi-structured questionnaire developed and validated by the World Health Organization (WHO) to identify the medical causes of death. (quantitive study).
	Study population and sample recruitment	-They conducted verbal autopsies at home among caretakers of the dead infants (162).<br>-Pregnancies and new births were identified through a doorto-door survey covering the entire community of 125,000 inhabitants, and pregnant women were followed up weekly until delivery. Infants were enrolled in the intervention trial at three weeks of age. Whenever an identified newborn did not turn up at the clinic for enrollment, a home-visit was made to identify the live/dead status. After the initial nine months of the study and until the 18th month, i.e. the end of enrollment, an informant-

		based system was used for identifying newborns or infants aged less than three weeks. During this period, deaths in the first three weeks of life were not ascertained.
	Data collection tools, process and analysis	-Female physicians, trained in ethnographic techniques, soon after the death but within 16 weeks, visited families of dead infants. They conducted verbal autopsies at home, using a semi-structured questionnaire developed and validated by the World Health Organization (WHO) to identify the medical causes of death. First, the narratives of the caretakers were recorded in the local language. During the narration, the caretakers were encouraged to describe, in details, the events in their own words pertaining to the illness and the circumstances that led to death. Following this, the structured component of the questionnaire for neonatal or postneonatal deaths was completed as appropriate. The questionnaire covered symptoms, signs, management of illness that led to death, and hospitalization details.<br>- Three paediatricians independently reviewed the completed verbal autopsy forms to assign up to four causes of death using the definitions provided with the verbal autopsy instrument for guidance. Disagreements between paediatricians regarding the designated causes of death were discussed together to arrive at a consensus. A paediatrician and a social scientist together reviewed the narratives to obtain an insight into the pathway to deaths. They focused on identifying points at which the death might have been averted, and the actions and behaviours that might have contributed to death. The inferences from this review were recorded in a structured format developed after a reiterative process involving several pilot evaluations. For deaths in the first week of life, standard medical definitions were used. The recommendations of Integrated Management of Childhood Illness (IMCI), proposed by the WHO, were considered the standard against which healthcare received by the infants as reported by their mothers during verbal autopsies was assessed.
	Findings relevant to the review	All caretakers reported full compliance with the prescribed oral drugs, injectables, and ORS. Four (40%) of 10 neonates and 18 (54%) of 33 older infants advised for hospitalization were taken to hospital. Common reasons for not complying with advice for hospitalization were the lack of perception that the child was gravely ill, perceived improvement in the condition of child, inability to leave the home because of other siblings, economic reasons, reluctance because of unpleasant past experiences, and advice against doing so by other family members.
5	Publication details	Das et al (2018) Patterns of illness disclosure among Indian slum dwellers: a qualitative study. *BMC International Health and Human Rights*. 18:3
	Main study objectives/research question	This study specifically aims to uncover the many facets of lay decision-making before future action is taken and the reasons underpinning illness-expressing behaviour among Indian urban slum dwellers. The research questions can be formulated as follows:To whom and to what extent do slum dwellers decide to disclose their illnesses? – What are the reasons not to disclose illness? – What are the reasons underpinning a delay in disclosing illness? – What are the reasons underpinning prompt disclosure of illness?
	Study area/ Country of study	The present study was conducted in four urban slums of India equally selected from two cities, Kolkata in east India and Bangalore in south India. The slums were purposively selected on the basis of intra- and intergeographical variation, age of the slum, religious plurality, variation in living conditions and the presence of different healthcare systems. The characteristics of the study areas, the Motijheel slum and SahidSmriti Colony of Kolkata as well as the Nakkle-Bande slum and UllaluUpanagar of Bangalore.

	Study design	This study is part of a larger qualitative enquiry investigating health beliefs and practices among urban slum dwellers.
	Study population and sample recruitment	This study included 245 participants equally selected from both slums, of whom 129 males and 116 females. From this sample, 218 participants (105 men and 113 women) reported to the researcher to have experienced illness during the 12 months prior to the interview. Their subsequent responses towards illness gave rise to three different patterns of disclosure, to wit prompt disclosure, delayed disclosure and no disclosure at all.2 These participants were purposively selected for this study and were subsequently interviewed to investigate their behaviour in relation to illness disclosure. During the pilot study, we realised that a six-month recall period was too limited to collect extensive data on reporting behaviour. We attained data saturation after two to three interviews, since many participants reported not having experiencing any major or minor health incident in the past 6 months. As a result, a oneyear recall period was used for both minor and chronic illnesses. A study by Kjellsson et al. also indicates that using a recall period of 1 year is preferable to scaling up a recall period of one, three or 6 months. According to Sudman et al., respondents are aware of the risks when being questioned on the recent past and prefer to disclose a more distant yet noteworthy event. As such an approach might influence their responses, it has been suggested as a way to reduce reporting errors. For multiple minor illnesses that occurred within a period of 1 year prior to the interview, the most recent case was considered for the study. The minimum age of the participants eligible for the study was fixed at 16 years, as it is assumed that the onset of puberty establishes self-awareness about one's body and health.
	Data collection tools, process and analysis	-In Kolkata, data on the health-seeking behaviour of slum dwellers were collected during August and October 2012. The main researcher personally conducted the interviews due to her familiarity with the local language (Bengali). All the respondents were told the purpose of the study, their part in the research, and the time and energy that they had to provide, as well as the possibility of pain, discomfort and stress that they might experience during the interview. Participation of the respondents was voluntary. Women were interviewed in their homes, while men were interviewed during their leisure period or sometimes in their workplace near their homes. The researcher audio-recorded the interviews and took notes. Each interview lasted for about 30 to 40 min. The Bangalore field study took place from July to December 2011. In-depth interviews were carried out in a conversational style with a semi-structured interview schedule for data collection. A female field investigator collected the field data under the guidance of the main researcher, as the local language (Kannada) was unfamiliar to the atter. Prior to the start of the fieldwork, the investigator received 1 week of guidance from the researcher to understand the scope and objective of the study, and to conduct mock interviews and pilot interviews. All interviews were either conducted in a separate room at the participants' homes or in a separate spot at their workplace away from other people, in order to safeguard the privacy of the participants.<br>- A semi-structured interview schedule was constructed according to the basic framework of Arthur Kleinman's Explanatory Model of illness (EM). The questions were of an open-ended nature in keeping with the emic perspective of this study, which aims to gather an in-depth understanding of the sociocultural context, respondents' perceptions and self-constructed meanings. The researcher had already established a rapport with the participants through frequent visits in the field, participation in their day-to-day activities, and the first session of interviews where we explored and discussed their perception of health. To understand the reporting behaviour of the participants, the interview schedule encompassed questions on perceptions of the concept of illness, the threshold for recognising illness, aetiology of illness, types of confidants and the type or nature of illness

		(minor, chronic, communicable, reproductive and sexually related ailments). Whether or not this information was shared underpins the causes of disclosure attitudes and the timing of disclosure. Probing questions were used for clarification if more information was needed to explore issues raised by the respondents or to confirm the validity of their answers through cross-checks. Prior to the interview, the researchers decided not to define concepts such as pain, discomfort or inability to perform duties, which are inherently socially constructed. As such, the same condition can trigger different levels of pain and discomfort between individuals or within the same individual at different points in time. One reason behind this choice is that explaining and defining such concepts to the respondents before the study would have failed to create a 'common understanding' of these constructs, because they remain inherently confined to the subjective sphere. Another reason is that such an attempt to quantify how respondents experience these constructs would have felt as an imposition of the researcher's point of view and violated the emic perspective of gathering in-depth, subjective, socially constructed perceptions.<br>-Recordings and field notes of the in-depth interview series were transcribed verbatim in their original Bengali and Kannada languages. Bengali and Kannada transcripts were then translated into English. One Bengali and one Kannada speaking person were hired for this purpose. The first researcher double-checked all data for unclear passages and potential translation errors. Passages that raised doubts were cross-checked by the first and second author and, if applicable, further discussed with the translator. Once the body of text deriving from the interviews had been cleaned up and processed, the data were analysed using the 'thematic analysis' method. This manual analysis adhered to the guidelines of Braun & Clarke [35]. Initially, the data were read through several times to ensure a thorough understanding. Patterns within the data were identified manually, coded and subsequently labelled according to their meaning. Codes sharing a common meaning were grouped under non-overlapping themes or categories. These themes were re-examined for further refinement by comparing them with the original statements in the participant's accounts of their experience or perception of illness, their propensity to accept illness and their attitude towards reporting.
	Findings relevant to the review	**Choice of confidants**<br>Theme 1 – Healthcare professionals as the final recipients of illness disclosure.<br>The choice of the confidants to whom illness is firstly disclosed is markedly different between men and women, as well as being dependent on the nature of the illness. Women involve different people to disclose their illness at different levels. For instance, women initially said that they prefer and feel more comfortable to discuss their health problems with family members. By family members, they meant natal relations, particularly mother and sisters. Although in-laws and other affinal kin are occasionally involved, this choice depends on the nature of the illness. Common health problems are generally expressed to in laws, but illnesses that lead to stigma, familial defamation, or negative and informal social sanctions are usually held back as long as they do not attract the attention of others. After natal kin, husbands are informed of illnesses the most often. Such reporting behaviour may find its roots in how families and society respond to and treat their illnesses. Women feel that natal family members are more compassionate, attentive and considerate than in-laws and their families. In this context, one woman said:<br>Since childhood, I have been very weak and I am frequently seized by health problems... My mother is my power; she will always encourage me by making me feel special; whenever I am ill, she will not allow me to work, she will give me hot food,

		remain awake the whole night to check whether I am okay or not...My mother-in-law will not understand that. Instead, she blames my mother for giving them a physically unfit bride.<br>Women in the study expressed that they experience severe anxiety and fear when it comes to female-related illnesses and communicable diseases which involve physical examination. Indian women are conditioned by multiple socially prescribed codes associated with sexuality surrounding marriage. For example, they are requested to maintain physical distance from men other than their husband, not to attract men by flaunting or exposing body parts and to protect their virginity before marriage. To keep things comfortable, women therefore report their health problems to informal healers, which marks the first step for them to report outside of the family. Informal healers are preferred to professionals, because the former generally reside in the neighbourhood and are well acquainted with all the households in the area, their health habits, practices and behaviours. As a result, respondents feel more at ease openly to discuss healthrelated problems at length. According to one woman:<br>I talk at length [with the informal healer]. I tell him exactly how I feel about my illness; if it sometimes sounds funny, still he listens, comforts, encourages and helps me to come to terms with the trauma.<br>Another reason for approaching informal healers in the study is women's perception of such illnesses as complex conditions, which creates the need for them to confide in people who have knowledge about the illness and are better able to understand their mental stress. As one woman said:<br>It's okay to talk about your problems with family members, but they cannot always comfort you; there are certain female-related problems which are complex in nature and need to be understood by some knowledgeable person who can understand our emotions thoroughly.<br>Reporting to formal healthcare professionals in case of health problems only happens when they become acute or severe. Women in the study expressed that professionals do not have the habit of allowing patients to share their views and distress about illnesses, or to give emotional and social support in order to cope with the illness. Rather, they conduct an immediate diagnosis and treatment whenever patients visit them. In this context, one woman recalls her experience:<br>I went to him [the doctor], not with the intention to start treatment immediately but first to share my suffering and feel relieved. I expected him to comfort me by saying good and positive things and to give me the mental strength to deal with the illness, but he was focusing more on treatment than on listening to me and my feelings.<br>This kind of distance which women perceive in relation to formal healthcare professionals influences their disclosure pattern, prompting disclosure to either family members or informal healers rather than to doctors. By contrast, men prefer not to discuss general health problems with anyone, because they consider these problems to be too normal to discuss. In case of chronic or serious health problems, however, they report to their wives first and to the health professionals next. Parents, close relatives and possibly people who are going through a similar phase are occasionally informed about an illness in order to receive support when men are overwhelmed by negative feelings. Most men report involving their wives mainly to receive emotional support when they are going through a difficult time. Some men said that they disclose illness to their wives because they know that wives would not speak about a 'husband's illness' to others, as this information will hurt both her and her husband's self-image. As one man said:

		She feels that my illness is her illness and so she respects my emotions by not raising the issue with others.<br>Outside of the family, men habitually hold back from informing friends, fellow employees and acquaintances. Many men found that when they tried to disclose their illnesses to these persons, their intense emotions were underestimated and their manhood was questioned. Showing emotions and weakness was considered as uncommon for men, with many of them expressing that it is very essential in a group of men to show off one's manhood even when they are experiencing immense internal turmoil. As one man said:<br>Whenever all us men folk gather in the evening, we discuss downright everything from poverty, politics and problems in the family to the workplace; however, everyone is conscious not to discuss one's health problem. No one reveals health problems, as this is the only place where men can show off their manhood and vigour.<br>Unlike women, men find formal healthcare professionals to be quite efficient in providing comfort during stress. Many men in the study related that they can express their most intense emotions when they talk to doctors. This experience is linked to the way that they deal with formal healthcare professionals. Most of the men said that professionals react normally when men pour out their emotional turmoil related to illness. Some men also expressed the sensation during their communications with professionals that doctors find it quite natural and obvious that patients talk about their problems, irrespective of gender. In addition, formal healthcare professionals find the perception of manhood as related to health quite funny and irrational. As one man said:<br>He [the doctor] started laughing and though it was a joke when I told him that I cannot express my feelings to friends in the same way as I did with him, because I will be labelled as a woman.<br>Theme 2 –social norms and fear of future social sanctions driving the choice of confidants.<br>The choice of confidants highlights a complex interplay of gender, status and the nature of the illness. For instance, many men and women who were interviewed expressed that young men and women, both married and unmarried, according to prescribed norms are allowed to talk about common health problems with immediate family (spouse, parents, children, uncles, grandparents, nephews, and so on), extended family (great-grandparents and other common ancestors) and family-in-law (parents-in-law and siblings-in-law) irrespective of age and gender. However, for specific illnesses such as female-related illnesses, sexually related illnesses and sexually transmitted diseases, discussion is not allowed between young and old or between genders when they share a liaison relationship such as brother and sister, father and daughter or father-in-law and daughter-in-law. As one woman said:<br>It is very embarrassing and equally unmannered if you talk about female problems even when your father-in-law is in the other room.<br>For other communicable or for chronic diseases, it is observed that no rules are prescribed. However, as these illnesses create penalties such as social exclusion, familial defamation and individual disgrace, participants said that it is they who create informal rules; for example, revealing it only to spouses or natal families in order to protect their honour. One man commented:<br>Society has not told us to suppress serious health problems, but we sometimes have to do so in order to secure our and our families' position in society.

		No rules have been prescribed for men in informing non-kin and professionals. However, as women's relationships with professionals and non-kin (other than healers) of the opposite sex are considered to belong to liaison relationships, they are not allowed to talk freely about sexually oriented illnesses. As one woman said:<br>People will look at you unfavourably if you frequently talk about sexual problems, even with doctors and other males.<br>It is observed that women's marital status and the nature of their illness also influence the way that illness is disclosed to the families. For instance, many unmarried women with abnormalities related to reproduction or imperfections of the body prefer an early disclosure of the illness. They consider marriage as essential to obtain both personal and financial protection. As it is their belief that society views them as vehicles to produce progeny, the chances of getting married are largely determined by how physically fit they are. Consequently, women and their family tend to ensure that any problem related to health is sorted first before it creates any obstacle to their marriage process.<br>One woman talked about severe consequences which her sister had to face because of an initial failure to disclose her illness:<br>She had been suffering from abnormal vaginal discharges since two years and had not informed anyone in the family, not even my mother. When my mother was finally told, she was shocked not so much by hearing about the illness but sensing that it may cause problems in her marriage...at that time, we were planning to get her married. My mother consulted a doctor who detected damage in her 'baby pipe' [fallopian tube].The doctor said that unless it is operated on, she cannot conceive. There was nothing we could do; the surgery would bring a huge expense that we cannot afford; everything was out of control. She remained unmarried, as we could not get her through [marriage] without telling the truth. Every alliance turned her down when they came to know the truth. There was no end to our problems. As time passed, we started to face new problems. As she is beautiful, she was naturally harassed often by some men from other localities. We even came to hear that there were men waiting for any opportunity to make a sexual move. It was becoming difficult for my parents to keep an eye on her every time and everywhere. As my sister started to feel unprotected, my father sent her to our village.<br>In the case of married women, a few said that they sometimes reveal their problem to the family in-laws immediately as well, with the intention to gain their support and confidence. By talking to their mothers-in-law, women aim to show them that they are equally concerned about producing progeny. Indirectly, they also manage to secure their own marriage and financial security.<br>One woman explained how this action worked positively for her:<br>I took my mother-in-law into confidence by talking to her. I expressed my struggle with intense emotions and how I coped with them in battling the guilt of not being able to give them a grandchild. I also told her that I will do whatever is required to conceive and that's a promise. By listening to my sorrows and pledge, her heart melted, and she started to sympathize. She told my husband that I have no wrong intentions; in fact, I am trying hard to give him a child. She also told him to take good care of me in every aspect and to be by my side during this turmoil. Now, as my husband can't disobey his mother, he is doing what he has been told. I am getting attention both mentally and financially. Of course, I am trying hard to sort the problem and give him a baby, but at least I can do so without the constant worry of being thrown out of the house and out of my husband's life.

Onuegbu C, *et al. BMJ Open* 2021; 11:e050766. doi: 10.1136/bmjopen-2021-050766

		It is equally essential for both men and women to take care of the way in which they relate their illness. Many participants expressed that time, place and person are crucial when it comes to sharing illness-related emotions. A woman said that they are not supposed to talk about illnesses on any auspicious occasions such as initiations, marriages, baby shower parties and religious ceremonies held at familial and social levels. As illness is considered a negative element, people believe that talking about it on joyous occasions and festivals will create an ominous effect.<br>Another man expressed his belief as follows:<br>Occasions and festivals are pious and holy moments where everybody is intent and bent on blessings, goodwill and affluence in life. As illness symbolises agony and sadness, talking about it on such occasions will damage the atmosphere and spread negative feelings which nobody wants.<br>**Reasons to delay disclosure**<br>Slum dwellers reportedly delay the disclosure of illness to family members, kin, non-kin and professionals because of various reasons. The main categories of reasons are bearable physical burden, negative prior experiences of illness and coping with the insecurity of the slum.<br>Theme 3– Bearable physical burden Most men in the study looked primarily at biological aspects as determinants for reporting, such as the level of physical pain and their capacity to bear it. Physical pains that are minor and that can be handled are considered by men as belonging to general health problems and are therefore delayed in reporting. They psychologically made themselves resistant to general illnesses and consider these to be very normal in everyday life, as is reflected in one respondent's attitude:<br>Minor pains can be handled... they are not that serious; cold and fever are like frequent guests... they come and go; for these, I do not need to tell everyone immediately.<br>As women in this case are far more sensitive, they are not seen to delay reporting. Although women do not intentionally delay reporting illness, however, a few of them said that they sometimes do so when they lack proper knowledge or information about the severity level or the effects of an illness. As one woman commented:<br>I was not having my period for several months and I was ignoring it [considering it to be a normal phenomenon]. My mother told me that it was not normal, as it can create a problem while conceiving, and disclosed it to our guruji [informal healer].<br>Theme 4 – Negative prior experiences of illness<br>For both men and women, expressing or reporting illness is related to their past experiences with the responses that they received from others. Illnesses related to unpleasant memories discourage slum dwellers from reporting when the same symptoms reappear. Men and women shared their experiences with unpleasant memories differently. Many men in the study stated that disclosing illness negatively affected their livelihood in the past. This situation meant that the frequent discussion of illness and the expression of feelings in the workplace signalled to others and to the authorities either the person's lack of interest in his job and his desire to leave on the pretext of illness, or an attempt to raise his pay by triggering others' sympathy in the name of illness. As a result, they had often been fired for displaying illness-related emotions and had had to bear a sudden financial crisis. One man described the difficulties that he faced when expressing illness in the workplace:

		It's just that I was talking about my illness to one of my colleagues for two consecutive days. My boss noted this fact and misunderstood it as if I was doing so intentionally for him to hear. The third day, he dismissed me from the job, saying that he can understand my feelings of not being satisfied with the job and therefore inventing the excuse of illness.<br>For women, unpleasant past experiences associated with reporting illness are more closely related to sociocultural context. Many women in the study found that too many negative discussions about their own illness with kin and non-kin result in exclusion from familial and social ceremonies.<br>As one woman said:<br>I used to tell all the negative things that I felt about my illness to my sister-in-law. Some months later, I discovered that she had not invited me to her baby shower ceremony. I was hurt. Later, I came to know from one of my distant relatives that she had been saddened and that she feared a bad omen. Therefore, she did not want me to be there during that auspicious ceremony. I know all that happened because of my talking too much.<br>**Theme 5 – Coping with livelihood and everyday financial struggle**<br>Both men and women commonly acknowledged that their livelihood and the everyday financial struggle which they face prevent them from promptly disclosing their illness to anyone. Instead of focusing on health and illness, the slum dwellers feel compelled to concentrate on securing the basic necessities of life. For instance, the struggle to retain their job plays a crucial part. Most slum dwellers work in the informal sector under the constant threat of losing their jobs. It is important for them to secure their job by making an extra effort and showing their commitment to the job, by meeting the daily or weekly targets and by remaining present onsite for long hours. Many men who were interviewed expressed that they do not consider illness important enough to be shared and discussed, as it will bring no immediate harm to their livelihood. Rather, it is their bread and butter which are primarily affected if they do not talk with others about the job market situation or think about better chances in employment. One man said:<br>My health will not take away my bread and butter if I do not think or talk about it for five days. But if I do not show my commitment to work and do not take it seriously for even one day, I have to think for the other five days what my family will eat.<br>Consequently, some men mentioned their attempts to divert their mind from thinking about illness or burdening it too much in order to secure their wages. As one man said:<br>If you talk about illness, this means you are thinking about it constantly, and thinking about it means you are actually not well. The next day, you take a leave from your work feeling very sick; and your one-day wage is gone. The moment you stop talking about it, you will find life is normal...it's all about your mind.<br>Closely related to this topic are financial struggles, which involve a lack of or a meagre family income, depletion or lack of savings, unemployment or underemployment, excessive debt and uncertainty about the future flow of income. All of these factors force slum dwellers to consider health as a secondary aspect. Some women said that they delay the disclosure of health problems to professionals because they find that the professionals are expensive. They fear that going to professionals early on will bring crises after treatment, such as selling or mortgaging property because of debt. On this topic, one woman commented:

		Forget about sharing things with him [the doctor]. Once you go there, he will immediately start his expensive treatment and you have to start selling everything for the treatment. The later you go, the better. At least then, the impact of the crisis will be less.<br>**Reasons not to delay disclosure** This theme illustrates the reasons that prompt the participants of the study to express or report illness. In many cases, the intention behind informing others about illnesses in time is related to finding solutions, coping with the distress or preventing potential collateral damage. The themes that emerge are unbearable discomfort, therapeutic value of disclosure, fear of unfamiliar illnesses and previous negative outcomes of non-reporting or delay.<br>**Theme 6 – Unbearable discomfort** Men report disclosure when the pain becomes unbearable or unusual, as reflected in one man's comment:<br>I have had enough of this leg pain and cannot bear it anymore. I reported itto a doctor immediately.<br>Some men noted that it is difficult for them to assess the intensity of illnesses which occur internally, due to a lack of knowledge related to human anatomy or body functioning. Any strange internal pain or discomfort that happens, even for the first time, is therefore experienced and labelled by them as severe and reported without delay. As one man said:<br>One day, I vomited three to four times. It happened to me for the first time. I felt something very wrong was going on inside. I got so scared that I immediately talked about it to one of the local doctors.<br>**Theme 7 – Therapeutic value of disclosure** Women are found to be very sensitive to illness or bodily discomforts and show more willingness to express symptoms of distress to others for psychological relief as compared to men. Sometimes, expressing distress rather had to do with the complexity of the female reproductive system and represented a coping mechanism and a deliberate act of self-encouragement. Most women consider female-related health problems as unavoidable because of the complexity of the reproductive system. Such belief has emerged from their socialisation process, as they have been told and made to believe since childhood that their biological composition is responsible for their morbidity and even puts them at risk of death. As a result, women developed the coping strategy of reassuring themselves by continuously expressing their distress to others. As one woman said, reporting to others actually helps women to comfort themselves that everything will be alright and that reproduction-related morbidity will not necessarily lead to death. The same woman mentioned the following:<br>We tell our distress only to hear from others about women whom they know, who led a healthy life and died due to old age. By listening to all these accounts, we actually try to create hope in ourselves that we can also live in the same way and die due to old age rather than from female-related problems.<br>Moreover, they share their health problems with each other to find out various possible solutions to similar experiences that they have had in relation to illness. Such sharing of experiences raises increased interest among the women who face challenges. One woman described how she benefits from the illness related talks with her friends:<br>Every evening, we sit and chat about our daily life experiences; talking about health is one of our favourite topics. We have come to know many unknown things from each other, and we share various techniques and methods to protect and prevent illnesses from occurring. This information seriously helps...at least, I am confident that I can come up with whatever is needed to protect me and my family.

Onuegbu C, *et al. BMJ Open* 2021; 11:e050766. doi: 10.1136/bmjopen-2021-050766

		Many women find that a failure to express or disclose one's illness even for a single day means allowing the illness to breed inside the body. They further feel that this approach will yield nothing positive but will instead bring unhappiness in life, as well as weakening one's immune system and strength to survive. As explained by one woman:<br>The sooner you pay attention to health problems, the better you feel. These things are not meant to be hidden; at least, they should be shared with family and friends the day you sense them. Otherwise, the distress will eat you up slowly, making you more miserable and lifeless.<br>**Theme 8 – Fear of unfamiliar illnesses** One of the major concerns raised by men and women in the study relates to their unfamiliarity with symptoms or their lack of knowledge with which to assess the severity of the illness. Symptoms that do not show similarities with common health problems such as heart conditions, lung problems or abdominal pains are difficult to interpret and therefore cause preoccupation. Such symptoms include the presence of any kind of blot or patch without pain, any unusual disfigurement or any prolonged unexplained illness. Many men realise that they very easily get anxious about such unknown symptoms and start creating a commotion within the family. As one man said:<br>I got very scared... the whole area around my genital parts was full of several abscesses...I only sensed that something terrible had happened to me and immediately informed my wife.<br>Some men said that they get puzzled and therefore feel the strong sense to report it to the professionals. One man who was suffering from cyanosis recalled his first reaction:<br>I was too confused to even think about it, I have never seen or heard such strange things happening to others. It was awful to see the colour of your skin changing. Without wasting any more time, I immediately asked a doctor for help.<br>Such non-delay in reporting is due to a lack of knowledge about the nature and severity of these unfamiliar symptoms whose effect cannot be predicted. A number of women in this study also experienced unfamiliar symptoms. They immediately disclosed them to the family but remained composed while dealing with the situation, as they are mentally prepared to accept and adjust to any kind of illness. As one woman said:<br>These things [unknown illnesses] are very dangerous; you never know how it's going to spill over. In this situation, you need to be very cool and calm in order to act wisely. Throwing tantrums over it will only make it worse.<br>**Theme 9 – Previous negative outcomes of non-reporting or delay**<br>As already discussed in an earlier section, the previous experiences of the participants relating to the expression of illness can become a major cause of their delayed reporting. However, this factor is an equally important reason for the timely reporting of illnesses. Such reporting patterns vary with the situation, though. In this context, for instance, the immediate reporting of illness is linked with unpleasant memories of the past where ignoring illness had made the participants face intolerable pain or serious damage to the body. Many men and women in the study related the unforeseen consequences that they had to face because of delaying disclosure or not reporting illness. One man commented:<br>Once, I fell off a truck. I did not find it that important to discuss with everyone. Within a month, I realised that I could not sit or stand straight. After consulting with the doctor, we discovered an injury in the spinal cord that had actually occurred during the accident. I was out of work for almost seven months and there was no earning in my house.<br>One woman said:

Onuegbu C, *et al. BMJ Open* 2021; 11:e050766. doi: 10.1136/bmjopen-2021-050766

		My right hand became paralysed and remained invalid for several years because of disregard. I felt that I was becoming a burden to the family-in-law, as I was of no help [in doing household chores]. They were angry that I had not at least informed them about my illness. They blamed me solely for my situation.<br>As a result, the participants are scared to take a second risk when they experience similar symptoms relating to the mishap and therefore report the problem immediately to the family or the professionals.<br>**Reasons not to disclose** It seems that men and women in the study sometimes deliberately withdraw themselves both emotionally and verbally from family and others. In this section, we are concerned rather with the explicit notion of avoiding to express feelings in relation to illness.<br>**Theme 10 – Withdrawal as a coping strategy** Most men noted that they try to avoid highlighting problems by deliberately downplaying their impact or severity. The function of this behaviour could be interpreted as the avoidance of difficult feelings by selfcoping with illness and looking for a way towards a normal life. One man described his strategy as finding ways in daily life to prevent illness from entering the mind:<br>I do not let any illness hover in my mind...I talk to others about the usual things rather than about illness, I play with children, do light work at home, play cards with friends in the evening and go to the local temple. These things help me to distract myself rather than getting preoccupied with illness.<br>Some men feel that telling others is equivalent to allowing emotions to grow, which further deteriorates their mental state. In this quest of finding a normal equilibrium in daily life as soon as possible, they therefore refrain from reporting illnesses to others. As one man said:<br>People will not let you forget your illness if you share it with them. They will constantly make you feel ill by asking every time you see them how you are feeling and what steps you have taken to deal with it. It’s a struggle with yourself and your will, so I find that it’s easier to make yourself feel normal by not letting others know about your illnesses.<br>**Theme 11 – Perceived threat to social image**<br>Although men do not have to bear social consequences to the same extent as women, they nevertheless remain under the pressure of gender-related societal perceptions where they are expected to be strong, tough and resistant to illness. As a result, men expressed that they find illness to reduce their status in the male hierarchy. For a man, displaying toughness represents a badge that they always have to wear, lest they lose their value and identity as a man in society. The more they bear pain and suffering, the more their degree of manliness increases. Many men consider masculinity as a symbol of strength, while talking about illness is seen as a ‘feminine’ thing. One man said:<br>We cannot show our health problems, you know...it’s a female thing to show weakness... we will become the laughing stock of our friends.<br>Another reason why men do not reveal their health problems in the family is linked with a shift in positions of power with women. As one man said:<br>I was bedridden for months and Razala [his wife] started taking me for granted in every aspect...I felt powerless. It’s really very difficult to accept that suffering from some health problem makes you invalid; your family, who once used to respect and listen to you, starts taking you for granted. I don’t want this to happen again, so it is better to keep the illness within myself.

		One man described the erectile dysfunction that he developed for some months as a consequence of ignoring his diabetes symptoms:<br>My doctor told me that it happened because of not checking my diabetes. Actually, I did not find it [the diabetes] that important to talk about...It was a blow to my manhood. I felt like committing suicide.<br>Theme 12 – Deteriorated atmosphere in slum environment<br>The slum dwellers occupy marginal positions in the sociocultural system of the community, inhabiting an unfit environment. In this respect, some male and female participants expressed that they struggled to cope with many slum-related practical issues such as environmental decay, poor infrastructural facilities, displacement, poverty and crime. All of these aspects severely undermined their sense of safety and security. Many of them described their daily struggle in relation to some of these issues. Prior to focusing on any health-related activities such as identifying, disclosing and treating illnesses, they believe that faulty basic infrastructure should be addressed such as improper toilet facilities, improper shelter, open drains or sewages and that basic needs should be satisfied including proper sleep, clothes and food. The feeling of insecurity when these basic needs are not met can overpower their attitude towards sharing illness. As one woman said:<br>If there are no proper water and toilet facilities, it is obvious that we will get ill...so there is no question of talking about illness and wasting time until and unless these problems get sorted.<br>Slum dwellers deal with life-threatening situation and sexual violence. As they have had to face the challenge of frequent displacement, they often become victim of theft, murder, molestation and rape. For women, securing protection during displacement and finding their way among various uncertainties hardly provide any room for illness. One man talked about his struggles during displacement and his constant fear, which do not leave any space to talk about illness:<br>When we all were thrown out from our previous location... We all came to a land that was already littered with waste and that was filthy. I somehow made a small, fragile tent to ensure my family's security; I had two adult daughters during that time and was worried about their protection. I used to spend sleepless nights keeping a watch on my daughters' safety. My son used to study in a nearby school free of cost, but he had to stop that too because of the distance. Even now, we live every moment in constant fear of being thrown out from this land as well. Now I am old and cannot struggle as before. After listening to all these things, do you think that we have the mental condition to sit and talk about illness? Whenever I suffer from any illness, I find a way out for myself. I do not have the habit of discussing it. Everyone here is busy fixing their own problems. So it's as though I neither have the patience to discuss illness, nor do people here have the patience to listen.
6	Publication details	Essendi *et al* (2010) Barriers to formal emergency obstetric care services' utilization. *Journal of Urban Health: Bulletin of the New York Academy of Medicine*, 88(2):356-69.
	Main study objectives/research question	The purpose of this study is to investigate poor urban Kenyan men and women's views on the factors that hinder the uptake of formal obstetric care services.
	Study area/ Country of study	The study settings are Viwandani and Korogocho, two informal settlements located in Nairobi, the capital city of Kenya. In these two settlements the African Population and Health Research Centre (APHRC) operates the Nairobi Urban Health and Demographic Surveillance System (NUHDSS) with about 60,000 registered inhabitants. The NUHDSS has monitored vital

		events like births, deaths, migration, and livelihood events of inhabitants of these two slum communities since 2001. Viwandani is located about 7 km southeast from Nairobi's city center and is bordered by the city's industrial area and the Nairobi River. It covers 0.52 km2 with a population density of 52,583 inhabitants/km2. Korogocho covers a smaller area than Viwandani (0.45 km2) and has higher population density (63,318 inhabitants/ km2). It is located about 12 km east of the city center and is on reserve land of the City Council. Compared with Viwandani, Korogocho has less population disparity with regard to sex and age distribution.
	Study design	This study uses qualitative data from a maternal health project implemented in 2006 in the Korogocho and Viwandani slums. The purpose of the project, which is part of a multi-country study involving the Kassena-Nankana District in northern Ghana and the state of Uttar Pradesh in India, was to provide a better understanding of the delays and barriers to emergency obstetric care utilization in low-resource urban settings in Nairobi.
	Study population and sample recruitment	From the NUHDSS database, all women aged between 12 and 54 years who had a pregnancy outcome in 2004–2005 were selected and interviewed. From this group, those who had life-threatening obstetric complications and failed to seek health care were purposively sampled and participated in focus group discussions. The complications reported were similar across the slums and age groups. They included abdominal pain, headache and swelling of the feet, high fever, blurry vision, prolonged labor, and excessive vaginal bleeding. Their partners, opinion leaders, traditional birth attendants (TBAs), and older women were also purposively sampled and participated in focus group discussions.
	Data collection tools, process and analysis	-In total, 16 focus group discussions (FGDs) were held with each of the groups, formed along similar socio-economic and demographic characteristics. Groups of women who had complications were composed based on demographic and slum residence status. Opinion leaders were selected in consultation with the chiefs.<br>-Six trained field workers (3 male and 3 female) conducted the interviews in Kiswahili.* The FGDs were conducted using an FGD interview guide. All the interviews were audio recorded and transcribed into English. The areas of investigation in this study included the respondents' perceptions of formal delivery care services and the barriers that the community experiences to utilizing formal obstetric care services.<br>-Transcribed Word files were imported into NUD*ST 6 software (QSR International Pty Ltd, Australia) for coding. This involved the continual reading of the transcripts and investigation of the themes emerging from the data for categories, linkages, and properties. In many instances, verbatim quotations were used to illustrate responses on relevant issues and themes.
	Findings relevant to the review	**Identification of the Danger Signs:** Reaching a decision to seek medical care when a woman experiences an obstetric complication begins with the ability to correctly recognize the symptoms and signs. Failure to correctly and promptly recognize the symptoms that require a birthing mother to be referred to a formal health facility could act as a barrier or a source of delay. Many participants in the discussions reported that some women in labor take a long time before deciding to seek help. One participant (Women aged 20–29 years) noted that: "I had labor pains for almost a whole week [presumably due to the embarrassment of seeking advice or help to visit a health facility from neighbors]. I got the pain from Sunday to a Thursday. I became very black. I started to rupture on a Monday and kept ignoring until later because I know of some neighbors who are just on standby to see how you will behave when you are in labor pain. You have to be strong so that you don't give them something

		to talk about.”. It emerged that a previous history of uncomplicated deliveries limit cause for alarm when complications start, contributing to a delay in deciding to seek help. Additionally, most women in the study areas were reported to seek delivery services from TBAs who live in the community, and TBAs are considered to be committed to the welfare of the community members and have earned their trust. As a result TBAs are considered key opinion leaders on birthing matters, and many women allow them to make decisions on their behalf especially if the husband, who typically considers himself the key decision maker, is unavailable. Many TBAs will promptly refer a case with complications, but in few reported cases the TBA insisted on handling the case, hoping that all would end well. As captured in the words of one TBA: “Some TBAs are also greedy. They keep the woman long past delivery time…they are supposed to refer such a case to hospital…” Also, although not widely reported, attributing curses to complications, also hinder prompt care seeking. **Poor Health Decision Making**: Correctly recognizing symptoms of an obstetric complication does not automatically translate into positive decision making to seek necessary care. Decision making emerged as a complex issue. Without a supportive spouse, family, or social network, the decision to refer a birthing mother with complications takes a long time to reach. Depending on the structure of decision-making power, the decision could either be made by the woman or any other relative, including the mother-in-law and the husband. Women who rely on their husbands for financial support may not be in a position to make referral decisions without their partners’ permission. TBAs reported that they sometimes make referral decisions on behalf of the mother when other family members are absent or when the mother is not in the right mental state to make this decision. In a few of the discussions it emerged that whenever the husband or other family member is uncooperative in decision making, the village chief or headman is sometimes involved
7	Publication details	Ghosh et al., (2010) A study on care seeking behavior of chest symptomatics in a slum of Bankura, West Bengal. *Indian Journal of Public Health,* 45: 1, 42-44
	Main study objectives/research question	To determine the prevalence of chest symptomatics among the study population, study their health care seeking behavior and identify the underlying socio-demographic correlates
	Study area/ Country of study	Patpur slum of Bankura Municipal area in the district of Bankura, West Bengal, the field practice area of the department of Community Medicine, B. S. Medical College, Bankura.
	Study design	A cross-sectional, community-based descriptive study was conducted during July to October 2008.
	Study population and sample recruitment	Persons aged 15 years and above residing in Patpur slum of Bankura Municipal area in the district of Bankura, West Bengal, the field practice area of the department of Community Medicine, B. S. Medical College, Bankura.
	Data collection tools, process and analysis	-A pre designed, pre-tested, semi-structured questionnaire in local vernacular was used. House to house visit was made for detection of "chest symptomatics'. After obtaining informed consent, relevant information regarding their socio-demographic characteristics and health care seeking behavior were collected by interviewing the subject concerned.<br>'Chest symptomatic' was defined as a person with cough for 3 weeks or more with or without haemoptysis, fever, chest pain, weight loss and / or night sweating. The study subjects were enquired whether such symptoms were present within a recall

		period of 2 weeks or not. The term 'Care seeking Behavior' meant action taken by the chest symptomatics for alleviation of their symptoms. Social class was determined by using modified Kuppuswamy scale (updating for 2007).<br>-Data were entered in MS Excel-2003. Proportion was used to calculate the prevalence of chest symptomatics and their health care seeking behavior.
	Findings relevant to the review	Initially, major reasons for choosing facilities were advice by family/ friends (43.8%), proximity to residence (29.2%), and expectation of better service (27%). For changing of facility, aspiration for better services was cited as most important reason by majority (50%) followed by advice of family/ friends (25%) and referral by the concerned facility (25%).
8	Publication details	Heijden *et a* (2019) Working to stay healthy',health-seeking behaviour in Bangladesh's urban slums: a qualitative study<br>*BMC Public Health* 19: 600
	Main study objectives/research question	The objective was to document how people perceive their health and care options and seek healthcare within this community.
	Study area/ Country of study	Dhaka City, Bangladesh, is one of the largest and fastest growing cities in the world. Approximately 35% of Dhaka's population of 15 million people are thought to live in slums, which continue to expand as Dhaka's population rises to an estimated 20 million by 2020. Kamrangirchar is the largest slum area in the Dhaka city. Combined with the neighbouring area of Hazaribagh, it has an estimated population of 485,000 residing in approximately 6.5km2 and is home to much of the city's informal manufacturing industry, including an estimated 150 tanneries.
	Study design	A qualitative descriptive explanatory approach was selected as most appropriate in meeting the study. objectives; We aimed to both describe health seeking behaviour in this context (what is going on), and to explain factors influencing it (why is it going on)
	Study population and sample recruitment	-As applied research directly linked to *MSF programming, our sampling strategy focussed on the population for whom these services were relevant, comprising women and girls aged 13–49years and factory workers.<br>*In 2013, Médecins Sans Frontières (MSF) started an urban healthcare programme in Kamrangirchar and Hazaribagh responding to unmet sexual and reproductive health needs amongst girls and young women aged 10–19years, and high rates of occupational illness and injury amongst workers in the area's small-scale factories.<br>-Women (13); factory workers (14); Purposive maximum variation sampling was used to identify participants with a wide range of perspectives on the study aims in order to identify important shared patterns emerging from a heterogeneous participant group [15]. Individual characteristics considered in participant selection were gender, age, profession, geographical location (indicative of predominant industry, e.g. metal, leather, plastic, garments) and perceived vulnerability (based on housing type). Recruitment of the female and worker participant groups was facilitated by MSF project staff, who approached a group of dwellings and requested the participation of an individual meeting the inclusion criteria.
	Data collection tools, process and analysis	-Data was collected through in-depth interviews, using a flexible participant-led approach based on a topic guide (Additional File 1). Interviews took place in a mutually agreed private location, usually the participant's home, and lasted for 60 to 90 minutes. They were conducted in Bangla or English, depending on participant preference, with JH and NG interviewing with the support of a translator. We ensured that the gender of the interviewers matched that of the participant. Interviews were

		audio-recorded, and recordings were transcribed and translated into English by experienced transcribers. Translators and transcribers received comprehensive training prior to data collection, as well as daily feedback from the researchers, JH and NG, to ensure careful contextual translation of idioms, metaphors, and other local expressions. Completed transcriptions were checked and a subset were back-translated by a second translator. Observations were written into field notes to support the interview data. Data collection continued until the team felt that no major new information was emerging from interviews and so theoretical saturation was reached, comprising 13 to 14 interviews per group.<br>- Data analysis was inductive and thematic using elements of phenomenological and grounded theory. This approach was selected as we aimed to generate new understandings of our study subject grounded in the views of our participants and articulated through a descriptive narrative. Open coding was used to break down, examine, compare, conceptualise and categorise data, followed by axial coding to 'put the data back together' in new ways, and selective coding to repeatedly apply core codes to transcripts (constant comparative analyses) toward the organic identification and development of patterns and themes emerging from the data. Codes were subsequently gathered in conceptual categories and organised into themes through a process of analytic reflection. Data were triangulated in order to maximise validity, and cases that did not fit with conclusions were re-analysed in order to test emerging theory and ensure that examples were not selected purely to reiterate desirable conclusions. Data analysis was conducted by JH using NVivo11, and a subset of data was coded and analysed by a second researcher, NG, to enhance reliability. Analytic memos were used to document the coding process and choices
	Findings relevant to the review	The majority of our participants obtained knowledge about health and health services through word-of-mouth. Information spread through the community as people shared their knowledge and ideas and learned from past experiences. Women particularly described the central role of their social networks to inform decisions about health, seeking advice from trusted female friends and family members. "Yes I [take advice from friends or family when I'm ill]. I think that's very normal. Whenever I don't understand the problem then I usually ask my friends or relatives to help me out. Sometimes I take advice from them about how to take the pills and medicines." P01 - MFW.<br>"I learn something from experience, like if someone caught fever or cold related problem they just take Napa tablet [paracetamol] for cure. Sometimes I also apply for this on my own problem. I saw my manager doing so when he feels sick; he just took some medicine like that." P16 - FFW.<br>For some, this information gathering process yielded contradictory advice, which increased confusion and uncertainty when facing numerous possible options for care.<br>"[I] ask people,'where is the best place for treatment?'. There are big brothers and sisters [that] I can ask about this. […] Problem is many people advise differently. So it is difficult to judge which decision is best." P16 - FFW.<br>These informal channels also posed challenges to accessing information on sensitive health issues, as participants mentioned feeling shy or uncomfortable sharing their symptoms with others. This was noted particularly for girls and younger women linked to sexual and reproductive health and family planning:<br>"Many girls cannot share what they should do after marriage due to shyness." P18 - HW.<br>"It is better if I get more ideas [about family planning]. As it was [my pregnancy] happened by mistake, so if I knew it earlier then I would've not made that mistake or won't make further. So I am in need of such ideas… I didn't have any idea [about

		family planning] before I got married. I didn't think of these things before... [After I was married] then also I didn't get any ideas or information. If someone would have asked me about this then I would pretend that I don't know about these things." P27 - HW.<br>Many participants explained that health decision-making within families had undergone changes over time, with responsibility and authority no longer held soley by the head of household as it had been in the past. For the majority of participants, however, health decisions were made collectively and informed by the opinions of family members, partners, and in some cases employers. This was particularly evident for women; those who were married generally consulted with their husband when seeking care for themselves or their children, and younger girls deferred decisions to their parents or older female relatives. This was often linked to the financial implications of the care choice, as those living separately from their husband or earning independently expressed more autonomy in terms of choosing and accessing care.<br>"There was once a time when the head of the family used to make decisions whether to go to hospital or not, will I take my son or daughter or wife to the hospital? There was once a time when we had to wait for his decision. Now the situation has changed. Now they are all getting to know from the welfare of different NGOs that they have this problem and they have to go to the hospital for treatment. Now the decision is being taken by every member of the family. Once it was like this that father-in-law used to be head of the family and to make decisions for taking to hospitals or not but now mother-in-law can also take the decision". P24 - KI.<br>"It can be different in different families, but many times it just happens that the husband or the earning person take the decision where people, where the family goes for their health." P26 - KI.<br>"It was my decision [to have an abortion]. I didn't say this to my husband or to my mother-in-law." P33 FFW.<br>For many participants, personal connections with care providers, as well as referrals from one trusted provider to another, often dictated the next step in a care-seeking journey. This was often linked to the perception that the referral would facilitate quicker, better quality or discounted treatment.<br>"My sister in law works [in the clinic] so we visit privately the doctor works there. So we are his people. My sister in law directly talks with the doctor, then she took me there, then she listens to me, then he gave me treatment, the treatment he gave we took them immediately…" P28 - FFW.<br>"[The pharmacist] gives medicines if he can. And if it is not possible for him then he suggests us to visit the hospital. At least I get ideas what to do in various situations… They refer some doctor in the hospital if they know someone closely. They provide me small notes or cards and say I might get discount, quick and good support on medical facilities if I show the small notes provided by the pharmacist." P01 - MFW. Participants explained that additional sources of health information comprised the many organisations going door-to-door, including people selling drugs and traditional remedies, and workers from various NGOs. Whilst some participants appreciated this effort, others expressed confusion at the different messages and services offered and mistrust in the motives of the workers.<br>"They go door to door to seek people and patients, they come to sell medicine… they say that they are doctors: 'don't mistrust us, we stay with the doctors, see – we have uniforms… we know what to give for what diseases, we don't want you to be sick,

		we want that you stay good, that's why we come door to door with care'. They come house to house just to keep their jobs..." P28 - FFW.
9	Publication details	Hu *et al* (2012)<br>Filipino women's tuberculosis care seeking experience in an urban poor setting: A socioecological perspective<br>*Health Care for Women International,* 33:1, 29-44.
	Main study objectives/research question	To characterize TB care seeking in Payatas and identify facilitators and barriers at the individual, household, community, and health-system levels from the perspective of the community.
	Study area/ Country of study	-Payatas has a population of 118,333 people, composed of three areas (Payatas A, Payatas B, and Lupang Pangako) and is built around a large open trash dump in Quezon City. Many households live in temporary housing made of salvageable materials, while few live in semipermanent or permanent structures. Individuals who scavenge trash for their livelihood usually live closer to the trash dump, while junk shop and store owners live farther away. Both men and women work to contribute to the financial security of their household.<br>-The Philippines
	Study design	We implemented the study in two phases, utilizing two types of qualitative methods: in-depth interviews (IDIs) and focus group discussions (FGDs).
	Study population and sample recruitment	First phase: Female patients who were receiving treatments from LGUs and NGOs. Eligibility criteria included being female, 18 years or older, resident of Payatas, and referred to TB diagnostics or a DOTS patient.<br>Second phase: Focus group participants were recruited through one of the participating NGO's microfinance saving groups composed of mothers.
	Data collection tools, process and analysis	-Female patients and community members<br>-Phase 1: We conducted a total of 22 semistructured IDIs with 13 female patients to understand their care seeking experience and identify barriers and facilitators to obtaining TB treatment. Eligibility criteria included being female, 18 years or older, resident of Payatas, and referred to TB diagnostics or a DOTS patient. For the informants who recently were referred to TB diagnostics, we captured an ongoing care seeking experience by conducting three in-depth interviews 2 weeks apart. One to two IDIs were conducted with the informants who were already enrolled in TB treatment.<br>Phase 2: We facilitated three focus group discussions from different areas of Payatas (Area A, N = 9; Area B, N = 6; Lupang Pangako, N = 14), to build upon the emerging themes from in-depth interviews and to explore community norms concerning attitudes and perceptions toward tuberculosis, care seeking, and the health system. During the discussion, we used a problem ranking exercise to understand the participants' rationale and decisions about the relative weight of barriers to care in comparison to one another. Participants were also prompted to offer suggestions on how health services can address these barriers. Each FGD was 60–90 minutes and facilitated by a field worker in Tagalog.
	Findings relevant to the review	**Individual level influences on care seeking:** Women illustrated the ways that fear of being diagnosed with TB was both a paralyzing and catalyzing factor for seeking health care. When asked what was preventing one mother, who has had symptoms

		of TB for 3 months, from following through on her referral, she distressfully responded, "I am afraid. I fear that I do have it. It's really fear that overwhelms me." Focus group participants also stated how nahihiya, or shame, came into play with fear: "The person would be scared that she would lose her job and that people and friends would avoid her." Fear about the consequences of TB, however, also pushed women toward care. One woman explained that her mother, a newly trained CHV, insisted that she ought to seek treatment, otherwise, her lungs would be "consumed" by TB. Fear and symptom recognition did not necessarily convince women they needed to seek care. One woman, who was referred to treatment, urgently explained that her neighbor's severe symptoms impacted her: "I was shocked that she [her neighbor] was already coughing blood. Then it was already too late. I will really do it [the negative direct sputum smear microscopy] this Thursday to do something before it's too late." Two weeks after this was proclaimed, however, she had yet to submit her sputum. A local CHV and her husband encouraged her to seek care; however, she told us, with frustration, that her household responsibilities inhibited her from following through. In her case, and many others, multiple barriers and facilitators were weighed in the treatment seeking process.<br>**Household level inflences on care seeking:** Support from family members and friends manifested itself in encouragement, being a kasama or companion, and taking on the mother's responsibilities. This did not just come from husbands, but also mothers, siblings, and adult children. One young woman, who lives with her in-laws, told of how her family members played a role in her care seeking:<br>"My husband said my cough has been there for a while, so he said that I should already have a checkup because my cough might not be normal. So Mama, my husband's mom, accompanied me to have a checkup." Women in FGDs also highlighted the importance of having a kasama, whether a family member or neighbor, by asserting that they would encourage and bring their neighbor to the clinic if she had TB. On the other hand, women who lacked a kasama to go along to a clinic expressed reluctance to follow through on referrals or get a checkup. Women voiced that unfamiliarity with the clinic's location and process made individuals fearful of accessing services. One woman jokingly stated, "I want someone to get lost with me." So she waited a few weeks until her neighbor, a CHV, brought her and others with referrals to an NGO in a different part of Payatas. Some women expressed that their husbands were supportive in the process, reminding their wives to find and finish treatment for the sake of the children. Often their husbands' encouragement also reflected their desire for their wives to contribute to the household finances. One woman was very grateful for her husband's support because he would "come home and cook because the children need to eat" instead of doing overtime at work. Another woman shared a fond memory of how her husband's humor and concern helped her overcome her initial response to her TB diagnosis:I went home [from the clinic] weak and pale then he got mad, "Why?" Then I said, "I have TB." Then I was crying and crying. "Crazy! You have TV!" So he made me laugh and I told him, "It's life threatening, right?" Then he said, "Crazy! Aren't you taking medicines?" I said, "Yes." "The reason why you're taking medicines is for that [TB] to stop!"' On the other hand, two women hid their symptoms from their husbands for different reasons. One woman feared being teased, while the other woman did not want to become a burden on her struggling family. Although, once the latter woman revealed her illness to her husband, he brought her to the clinic and told her fearfully, "If you had told me, I would have it treated earlier. So from now on, don't hide anything from me, whatever you feel. What if something happens to you?"<br>**Community level influences on care seeking:** Payatas is a squatter community in which "the houses are right next to each other,

Onuegbu C, *et al. BMJ Open* 2021; 11:e050766. doi: 10.1136/bmjopen-2021-050766

		and there are times when someone would find out that someone has something." Neighbors often work together, share meals, and watch each others' children. Women illustrated that within the community, neighbors treated people who were associated TB or TB like symptoms differently. According to mothers in the FGDs, neighbors would "be disgusted with you," "tease you" or "avoid you," if you have TB. Therefore, acquiring a TB diagnosis was a matter of great shame. These community norms toward those who have TB or TB-like symptoms influenced women's decisions to seek treatment. During an interview, one woman painfully recalled how her neighbors embarrassed her: "It's really shameful, especially when I'm outdoors, I cough a lot. They [her neighbors] would say, "That's already TB! Go to the Good Doctors (a NGO, pseudonym) now...." I am ashamed to say that I have TB, so I don't want to have a checkup." For her, not getting a checkup left her symptoms undefined, allowing her to say, "I really don't know what is it, if I do have [TB] or I don't, just symptoms." Focus group discussion (FGD) participants also confirmed this notion when they ranked nahihiya, or being ashamed, as an important barrier to seeking care because one "would think of what others would think [about them] before seeing a doctor." Furthermore, once people acquire a diagnosis of TB, they feel they need to abide by a different set of rules for interacting with other people, such as separating their food and utensils or avoiding social interactions. Women participating in focus groups and interviews alike expressed that these social norms can be ostracizing. Last, a few TB patients and FGD participants thought that TB is hereditary. Therefore, people did not want neighbors to associate their families with having TB. For example, when one woman revealed her sickness to her husband, he asked defensively, "Where did you get it? It's not from our family." Women eagerly asserted that their community's response to TB needs to change to encourage others to seek care. One FGD participant pointed out to her peers that when "someone understands your [TB] condition, she shouldn't stay away from you [and] instead encourage you that there's a cure and to get treatment; and one more thing, all of us will get sick, it's just going to be different kinds." Women with TB also proclaimed that they wanted "to become a proof to people that one shouldn't be ashamed of this sickness because it can be cured."
10	Publication details	de Zoysa *et* al (1998) Careseeking for illness in young infants in an urban slum in India. *Social Science & Medicine,* 47: 12, 2101-2111
	Main study objectives/research question	This study focused on assessing maternal recognition and interpretation of illness in young infants, and on identifying constraints to the adequate provision of care for the illness.
	Study area/ Country of study	The study was conducted in a slum settlement in New Delhi, which accommodates about 60,000 persons who have emigrated to the capital from various parts of India over the past two decades. The slum covers an area of about four square kilometers within a larger urban complex, and is very dense, comprising a tortuous network of narrow, winding lanes lined with open drains and flanked by mud and brick dwellings.
	Study design	Approaches to data collection and analysis were guided by those used in the focused ethnographic studies on acute respiratory infections developed by the World Health Organization (Gove and Pelto, 1994).
	Study population and sample recruitment	In-depth interviews were also conducted with 37 mothers of young infants (between one week and two months of age). Mother-infant pairs were identifed through conversations with slum inhabitants in the lanes, or with the help of community-based

		informants, such as key informants and field workers from the nongovernmental organizations. Repeated visits were made to some particularly responsive key informants and mothers.
	Data collection tools, process and analysis	-Narratives of illness episodes were sought from mothers who complained of a recent or current illness in their young infant. Nine episodes of illness were followed prospectively through repeated household visits.<br>-Approaches to data collection and analysis were guided by those used in the focused ethnographic studies on acute respiratory infections developed by the World Health Organization (Gove and Pelto, 1994).
	Findings relevant to the review	Mothers in this community are not oblivious to signs of illness in their young infants, and are not passive in finding ways to seek care for them, despite the hardship and deprivation that threaten all other aspects of their lives. The analysis of illness narratives indicates a high level of use of available health services. Mothers generally took prompt action in seeking advice for any problem in their young infant that they judged to be more than a temporary nuisance, especially if it led to a change in the young infant's behavior or breast milk intake. They also sought advice if they were uncertain about the infant's condition. As expressed by one mother: `We seek care if the problem is above our heads'. Home-based remedies were rarely used, except in the case of zukam (akin to the common cold), and of khans| (cough) which led to a number of treatments, mostly designed to warm up the baby, such as whole body massages with mustard oil and garlic, hot dry poultices and sun baths, or the administration of small amounts of home-made mixtures prepared with spices and a little breastmilk. Home-available or pharmacy-purchased drugs were not given to a young infant without a prior consultation with a health provider. Careseeking was usually very prompt, once it was felt to be necessary. Often, a young infant who developed a fever at night would be taken to a practitioner the next morning, and one who developed a fever in the course of the day would be taken to a practitioner that very evening. In a few instances, nonetheless, delays did set in, usually because the mother was unable to make arrangements for the care of her other children, or because she felt she should wait for her husband to come home to help out with advice or cash. Usually, however, women would turn to their neighbors in such instances for support with child care or for small cash loans.<br>Case study: "Sunil is the eighth born. His mother, Kamla, has lost three previous children due to sukha (two aged between one and two months, the third aged about two years). He was born at home, `strong and healthy'. He started a fever on the evening of the sixth day. His mother explained that she had caught a cold, with fever, because she slept under the fan, and that she passed it on to Sunil through her milk. He was upset, crying a lot, and wouldn't feed. The next morning his parents took him to Dr. A., a well-known RMP, whom they felt had a lot of experience. Dr. A. gave them two syrups. The next day his parents took him back for a follow-up visit and were reassured that his temperature was down. When the researchers visited the household for the first time a week later, Kamla expressed concern, because she felt that Sunil was getting thin, even though he was feeding well. She was given some breastfeeding advice and urged to take Sunil to the well baby clinic run by a local nongovernmental organization. Kamla did not visit the clinic. When Sunil was about three weeks old, he `dried up' and developed sukha. He also had loose stools, and was not feeding well. Within two days, Sunil's parents consulted another private practitioner, Dr. B. (an MBBS doctor), on the advice of a neighbor." Dr. B. prescribed some vitamin drops. The researchers made a repeat visit to the household that week. They noted that Sunil had visibly lost a lot of weight, and decided to take him and his parents to consult the physician (Dr. C.) from the vitamin A research project. On examination, Dr. C. found no signs of infection. However, Sunil's axillary temperature

Onuegbu C, *et al. BMJ Open* 2021; 11:e050766. doi: 10.1136/bmjopen-2021-050766

		was low and he weighed 1.7 kg. Dr. C. referred him to hospital for further assessment of suspected septicemia. Sunil's parents were distressed, but hesitant. In the end, they agreed to go with the researchers to the government hospital. On arrival at the hospital, they received a very rough and rude reception by the medical sta€ in the triage area, but were allowed to move on to the pediatric ward. There, after an hour's wait, a physician conducted a thorough examination of Sunil. She explained that he couldn't be admitted because he was so weak. She gave advice on breastfeeding, and prescribed an ayurvedic tonic to be taken by Kamla. When the researchers visited the next day, they provided an oral antibiotic for Sunil's use (on Dr. C.'s recommendation). Three days later, on the researcher's next visit, Sunil's father expressed helplessness: `We have seen all these doctors, and still he is not getting better. What are we to do now?'. The researchers took Sunil and his parents to consult Dr. D., a pediatrician in private practice nearby, who started him on intramuscular antibiotics, and subsequently recommended introducing bottle feeds, which Kamla did. Over the next four weeks, Sunil was taken back by his parents to Dr. D. seven times, and eventually he gained weight and became more active. All along, Sunil's parents expressed uncertainty about the causes of the illness. They didn't know why the other children had got sukha and died either.
11	Publication details	Taffa, *et al* (2005) Child morbidity and healthcare utilization in the slums of Nairobi, Kenya. *Journal of Tropical Pediatrics.* 51(5):279-84.
	Main study objectives/research question	The current study aims to assess determinants of child morbidity and healthcare utilization among slum residents in Nairobi City, Kenya.
	Study area/ Country of study	-The Nairobi Urban Demographic Surveillance System (NUDSS) pilot study was caried out in four slum areas of Nairobi namely, Kawangware, Korogocho, Njiru and Viwandani.<br>-Kenya
	Study design	The Nairobi Urban Demographic Surveillance System (NUDSS) pilot study was caried out in four slum areas of Nairobi. A total of seven rounds of surveillance were done and visits to the households were made every 90 days.
	Study population and sample recruitment	-Children in whom morbidity was reported at least once during the 9-month observation period.<br>-A total of 696 children below 5 years of age were registered during the 9 months period of follow-up. Males were 360 (51.7 per cent) and females were 336 (48.3 per cent). Of these, 264 (37.9 per cent) were reported to have been sick/injured at least once
	Data collection tools, process and analysis	-Data on demographic (births, death and migration), health (morbidity and verbal autopsy) and livelihood were collected on a regular basis. This paper presents analysis of child morbidity data. Information on child morbidity was collected for all children who got ill or injured since the last time a visit was made to that household. Details about child morbidity (illness/injury) including the signs and symptoms, the perceived diagnosis, and treatment seeking behavior were collected using child morbidity interview questionnaires adopted from UNICEF multiple indicator cluster survey (MICS2) and the WHO integrated management of childhood illness (IMCI).

		- Statistical analysis was done using SPSS10 for Windows program. w2 and odds ratios (OR) and 95 per cent confidence intervals (CI) for statistical associations were calculated for risk factors and outcome variables such as morbidity and health seeking behavior
	Findings relevant to the review	Health-seeking behaviour: This variable was assessed for the 264 children in whom morbidity was reported at least once during the 9-month observation period. Information on health seeking was considered for analysis during the first-time child morbidity was reported since there were on average more than one episode of morbidity for every child. Cough, fever, diarrhea, skin problems and eye diseases were the five top morbidities. Medical personnel (working in public or private sector) made the diagnosis of child illness in 51.5 per cent of the cases followed by family members (mother, father and others) who made diagnosis in 44.7 per cent.
12	Publication details	Uzma, *et al (*1999). Postpartum health in a Dhaka slum, *Social Science & Medicine*, 48, 313-320
	Main study objectives/research question	To describe the circumstances of women following childbirth by exploring patterns of birth-related illnesses, their health-care seeking behaviour and their beliefs and attitudes relating to both their illnesses and any services they have received.
	Study area/ Country of study	-This study was conducted in the slums of four wards of Motijheel thana in Dhaka city.<br>-Bangladesh
	Study design	Qualitative
	Study population and sample recruitment	-The main study population comprised all women who had recently experienced child birth and who were living in the study area. In order to obtain a more complete picture of the health related issues for women in the study area, focus group discussions were held with other women in the bostee. The third group of respondents from whom information was sought was health care providers. All health care providers (both allopathic and traditional) who the postpartum mothers reported as having utilised, were listed from the interviews. The seven providers women most frequently reported using were then interviewed using an unstructured interview guide.<br>The main study population were recruited during the first part of the study by a house to-house survey which identified all recent births and all women in the second half of pregnancy likely to deliver and reach 6 weeks postpartum before the study concluded. Imminent births to potential study women were recorded by research assistants, after which the principal investigator returned when each woman had reached approximately 6 weeks postpartum. These mothers were then interviewed using a semi-structured questionnaire. Those reporting health complaints were subsequently examined and offered treatment or referral as appropriate. In order to obtain a more complete picture of the health related issues for women in the study area, focus group discussions were held with other women in the bostee. The focus groups also allowed points raised in individual interviews to be clarified and enabled triangulation of information obtained from other sources. One focus group was held in each of the four wards of the bostee. Eight women aged 50 years or less who had given birth at least once, and who were living in the study area, were selected by systematic random sampling from women identi®ed during the survey as ful®lling these criteria. All the women selected agreed to participate in a focus group discussion.

		The third group of respondents from whom information was sought was health care providers. All health care providers (both allopathic and traditional) who the postpartum mothers reported as having utilised, were listed from the interviews. The seven providers women most frequently reported using were then interviewed using an unstructured interview guide.
	Data collection tools, process and analysis	-A range of research instruments was utilised in the study, including interview schedules, observational checklists.<br>-By incorporating a mix of qualitative and quantitative data from a range of sources, we endeavoured to provide a more complete picture of maternal morbidity and the dynamics of heath care utilisation.
	Findings relevant to the review	While the mothers used providers frequently, the actual process of seeking care was not straightforward; it involved multiple steps and was influenced by factors such as the gender of the provider, cultural traditions, perceptions of the causation and the severity of the illness, and the accessibility of care and cost of treatment. Broadly, the steps to consultation were first, making a decision about whether help from a practitioner was needed, second, selecting a practitioner, and third, actually seeking help. A general pattern to health care seeking was apparent: perceived mild and self-limiting illnesses were dealt with by a wait-and-see attitude; perceived serious problems usually resulted in the seeking of some form of care, usually traditional; and where the condition did not improve a second practitioner was usually sought, at this stage most commonly a western practitioner. This pattern varied for a range of reasons including the degree of trust a woman and her family had for a particular practitioner, the cost or potential cost of care, the acuteness of the problem and the perceived cause. The following case study highlights several of these issues.<br><br>3.1. Kulsum's story<br><br>Kulsum was born to a landless farming family and, like many girls in Bangladesh, was a thin teenager of just 13 years when she was married in 1981. Her first child was born in 1983 in her husband's village but died immediately after birth. Pregnancies followed in rapid succession and through each of them, Kulsum's health deteriorated. The fatigue from which she suffered was blamed upon asor (bad spirit). She was treated by kobirajis (a herbalist) and by mowlanas (a spiritual person), and was provided with amulets and holy water for her weakness.<br>Following a move from the village to the slums of Dhaka in 1992, and during her sixth pregnancy, Kulsum visited a western health care provider for the first time. She was 24 years of age. Diagnosed with pulmonary tuberculosis she was referred to the Dhaka Chest Hospital for treatment. Despite her own apparent willingness to receive treatment, Kulsum's husband could not afford to pay for 18 months of medication. He also expressed distrust of doctors in large hospitals. Furthermore, he blamed his wife for her health problems. ``I think she was entirely responsible for what happened to her. She never listened to me or my mother. Her movements during pregnancy were wild and that's how she attracted evil spirits which caused her all this trouble."<br>Kulsum's husband, however, did take her to a homeopath whom, he said, charged very little and gave her medicine for tuberculosis. She delivered her sixth baby at home with the help of a TBA. Three months later, Kulsum was pregnant for the last

		time. As the pregnancy progressed she became very thin, and suffered from breathing difficulties, chest pain and fever. The swamp nearby, which was used for the disposal of placentas and hence attracted bad spirits, was thought to be the cause of her illness. Labour began at 37 weeks, by which time Kulsum was almost bedridden. Very quickly she lapsed into unconsciousness. It took half an hour, in the back of a baby taxi, for Kulsum to reach the hospital. She died within ten minutes of her baby being born. She was 27 years old and left five surviving children.<br>Making a decision about whether a care provider should be consulted for illness during the postpartum period was dependent on cognitive, socio-demographic and economic factors. In broad terms, the decision making process began with the mother recognising certain symptoms which would compel her to seek outside care. In most of the cases in this study, respondents' husbands decided whether or not treatment from a practitioner would be sought, and in a small number of instances the respondents' mother or mother-in-law was the decision maker. Based on the study women's reports, it appeared that their di• culties were often ignored or minimised by husbands and by other family members involved in decision making.<br>Unless severe, symptoms associated with breast problems, perineal injuries, weakness, fatigue and vaginal bleeding were described by the women as essentially a normal consequence of life as a woman, and something they had to put up with. Either no treatment or home remedies were used for these conditions and assistance outside the family was generally not sought.<br>The process of selecting a practitioner was also complex. Potential cost was of central importance as these women were universally extremely poor. In addition, in this society, young married women without children, or with only small children have little power and hence even more limited access to the resources that are available (Blanchet, 1987). Apart from this often overwhelming problem there is the extent of the social distance between a very powerless woman living in a bostee and health professionals. It is therefore not surprising that cheaper traditional practitioners who live nearby were often the first choice.<br>Despite most illness being attributed to supernatural factors by the women, a surprising amount of non-traditional care was sought which suggests that these women and their families were prepared to try a range of alternatives. This readiness to accept western type medical care if it is affordable and accessible was confirmed by the very positive response to the investigator when she offered assistance with the women's health problems.<br>Once the practitioner had been selected, how care was actually obtained varied. The main constraining factor was whether or not the practitioner was available within the bostee. All the qualified practitioners and most of the unqualified western practitioners were located outside the bostee, which made it difficult for the women to approach them. Therefore, it was often a member of her family, usually her husband, who attended the practitioner, described the symptoms, obtained and took back the treatment to the patient in what is known as a `distant consultation'. Only eight respondents consulted a western practitioner in person. On the other hand, most TBAs and spiritualists were available locally and these practitioners were mostly consulted in person by the women. The herbalists and homeopaths lived outside the bostee and again with these practitioners distant consultations with the husband were the norm. In summary, access to health care of any sort is very difficult for these women for a combination of economic and cultural reasons. Their husbands generally controlled the finances and for practitioners not living in the same bostee, they controlled the entire decision making process. While the women were able to recognise important symptoms such as pain, bleeding and fever, they had little control over the decision making, and thus were forced to accept what was offered.

13	Publication details	Waghela *et al* (2018) Morbidity Pattern and Role of Community Health Workers in Urban Slums of Durg and Bhilai City of Chhattisgarh. *Indian Journal of Community Medicine* 43(3): 229–232.
	Main study objectives/research question	-To assess morbidity pattern and health-seeking behavior in urban slum hamlets of two cities (Durg and Bhilai)<br>-To understand the role of Mitanins (community health workers) in health seeking of their slum population.
	Study area/ Country of study	-A cross-sectional study was undertaken in a total of ten urban slums, five each from Durg and Bhilai, between June and July 30, 2017. Durg and Bhilai city are major industrial zones of Chattisgarh and hence selected for the study.<br>-India
	Study design	A cross-sectional study was undertaken in a total of ten urban slums, five each from Durg and Bhilai, between June and July 30, 2017.
	Study population and sample recruitment	-Data were collected through household survey. One resident, preferably an adult of each household in the hamlet was explained the nature, purpose, and objectives of the study.<br>-To attain maximum sample size, data collection from 500 households was estimated for each city. Average number of households in each urban slum hamlet of the cities vary from 75 to 150. So, in order to cover 500 households five urban hamlets were selected for the study. A total of 1025 households representing 4997 family members were surveyed. For sample selection, cluster sampling method was used. An urban primary health center (UPHC) of each city was selected through simple random sampling. Five slums falling in the catchment area of the UPHCs were selected randomly. Names of five hamlets for each city were drawn using the computer-based application.
	Data collection tools, process and analysis	-Data were collected through household survey. One resident, preferably an adult of each household in the hamlet was explained the nature, purpose, and objectives of the study. The person willing to be enrolled as a study participant was interviewed using the prestructured questionnaire. Informed verbal consent of the Mitanins and respondents from every household was sought before the data collection.<br>- The study variables employed to understand the morbidity pattern in urban slums were number of people with symptoms or diagnosed conditions in the past 15 days, number of people suffering from any chronic disease, that is, disease with more than 3 months of duration. Variables used to understand health-seeking behavior of the population were treatment-seeking behavior; contact with Mitanins; referral to public health-care facilities including subcenters, UPHCs, and district hospital; and referred to private facilities or traditional healers.<br>- One-fourth of the women respondents were illiterate in both cities. Less than one-fourth had completed secondary level of education in Durg, whereas slightly more than one-fourth had completed so in Bhilai. A quarter of the population in Durg and more than half in Bhilai did not have water supply sources at their houses. They had to use the community water supply source to get drinking water. 85% and 61% of population from Durg and Bhilai, respectively, had private toilets at their houses.
	Findings relevant to the review	In slums of Durg city, only one-fourth of the population came in contact with the Mitanins(in order to help us understand who these are I have copied the following texts from the background of the study: Mitanins are women volunteers selected by the community. Their role is to undertake family outreach services, community organization, and social mobilization on health and

		its determinants. The roles and responsibilities assigned to the Mitanins under the Mitanin Programme are to promote health, provide preventive health care to the community, treat minor ailments, health education, and referral. They act as the main link between the community and the public health system), whereas in Bhilai, slightly less than one-fourth came in contact with the Mitanins after feeling ill. People came in contact with Mitanins mainly to get their advice and help to reach health-care facility for treatment. Table 2 illustrates the conditions, for which advise from Mitanins were primarily sought. It shows that most people who came in contact with Mitanins went to public health facilities. Common health problems in relation to which people came in contact with Mitanins were chronic communicable disease and RCH related. Very few people suffering from acute diseases and chronic non-communicable conditions sought advice from Mitanins.

Onuegbu C, *et al. BMJ Open* 2021; 11:e050766. doi: 10.1136/bmjopen-2021-050766
